# “Trying to develop a better workforce”: Stakeholders’ perspectives of a practice-integrated Australian hospital pharmacist foundation residency program

**DOI:** 10.1371/journal.pone.0270051

**Published:** 2022-06-21

**Authors:** Yu Ting Sim, Carolyn Murray, Sally Marotti, Saravana Kumar

**Affiliations:** 1 SA Pharmacy, SA Health, Adelaide, South Australia, Australia; 2 Allied Health and Human Performance, University of South Australia, Adelaide, South Australia, Australia; 3 Clinical and Health Sciences, University of South Australia, Adelaide, South Australia, Australia; University of New South Wales, AUSTRALIA

## Abstract

**Background:**

Practice-integrated education and professional development programs (also known as residencies), have been available to pharmacists in America and the United Kingdom for many years. In 2016, the Society of Hospital Pharmacists of Australia launched Australia’s novel Foundation Residency Program to support the development of early-career pharmacists, and has been implemented across many hospitals nationally. This model was adopted by the South Australian (SA) public hospital pharmacy statewide service and was granted full accreditation. The study aimed to explore key stakeholders’ expectations and early perceptions of the structure, role and impact of the SA program and in that process, to identify key influencing factors and strategies informing future program planning and design.

**Methods:**

Purposeful sampling was adopted to recruit participants who oversee preceptors and residents, across all employment levels and pharmacy service sites. Stakeholders participated in individual semi-structured interviews. Each interview was audio-recorded and transcribed verbatim. The transcribed dataset was managed using NVivo software^TM^ (version 10) and analysed using reflexive thematic analysis through the lens of the PRECEDE-PROCEED logic model framework.

**Results:**

Thirty-three staff consented to participate. Participants were de-identified with a randomly assigned code number. Three key themes were identified using reflexive thematic analysis; alignment of program goals and visions, culture shift to prioritising workforce development as core business, program structure supports focused workforce development.

**Conclusions:**

Participants view the residency as beneficial for development of the residents, preceptors, and the hospital pharmacy workforce. The multisite structure was a strength of the program. Whilst it was acknowledged that the rotations, cross-site rotations, and research project presented challenges, they were deemed worth the investment. Overall, it was felt that incremental increases in program capacity will occur over time, as culture changes, and as investing in workforce development becomes core business. The findings have led to several key recommendations to guide program expansion.

## Introduction

Healthcare delivery demands a human health workforce, core to every healthcare system globally. The World Health Organisation [1] Global Strategy on Human Resources for Health reaffirms the pharmacy workforce as a key player in the healthcare team and as experts in the development, quality use and management of medicines. Safe effective access and use of medicines are fundamental to the prevention, diagnosis, treatment and cure of diseases [2]. Pharmacy practice is evolving, shifting from solely dispensing to medication management, therapeutics and direct patient assessment [3]. The key to optimising capacity for delivery of quality pharmaceutical services lies in a sustained investment in workforce education and training, leading to adaptable, flexible and proficient pharmacists. Evidence demonstrates that quality workforce intelligence is linked to improved health outcomes, employment and economy, ultimately strengthening health systems worldwide [2,4]. One of the essential global visions outlined in the 2016 International Pharmaceutical Federation report includes clear and intentional education and training frameworks that link competency-based and preceptor mentoring structures to complex practice-integrated experiential learning [4]. Practice-integrated frameworks such as a residency program, are well established in the United States of America and the United Kingdom [5–8].

In Australia, entry to workforce typically involves an undergraduate Bachelor of Pharmacy (or equivalent) or a graduate-entry Masters of Pharmacy accredited by the Australian Pharmacy Council. The new graduate is then required to undertake a one-year accredited structured workplace internship or pre-registration program. Full pharmacist registration is granted upon passing a national board examination [3]. Beyond registration and during the early career development and transition phase, there is a lack of structured practice-integrated education and development pathways. This poses a critical barrier to strengthening the roles and practice scopes of pharmacists nationwide. Typically, further career development is optional and self-directed outside the workplace either through an accredited Advancing Practice credentialing or postgraduate qualifications [3]. In 2017, the Society of Hospital Pharmacists of Australia (SHPA) achieved a national milestone by launching the first foundation residency program made available to all hospital pharmacies via an optional application and accreditation process. The program modeled a two-year practice-integrated training framework incorporating structured and mentored rotations within the hospital pharmacy setting. The residency program targets early career pharmacists (ideally within 1 to 3 years post registration) or those who are new to the hospital sector, and incorporates assessments and broader skill development mapped against the general level competencies of the Advanced Pharmacy Practice Framework [9].

In 2017, the South Australian public hospital pharmacy statewide service received provisional accreditation and the program was initially adopted by 2 out of the total of 5 local health networks. The program expanded state-wide in 2018 and was granted full SHPA accreditation in 2019. Residents consist predominantly of pharmacists either in their post-registration foundation years or relatively new to hospital pharmacy entering from non-hospital sectors, and are recruited from the pre-existing employee pool. The residency program consists of 4 core rotations over 6 months each, feedback-centered assessments, a research project and a local university-affiliated postgraduate qualification. At the end of the 2-year program, residents graduate with an accredited SHPA certificate as well as a Graduate Certificate in Clinical Pharmacy. The South Australian Pharmacy Foundation Residency currently represents Australia’s only state-wide multicentered program and with an integrated university-affiliated post-graduate curriculum. Resident pharmacists are recruited via a selection process with a limited pre-determined resident quota.

At present, there is only a single Australian publication of a qualitative study that explored stakeholders’ perspectives of program adoption nationwide which subsequently informed the diffusion and ongoing maintenance of pharmacy practice-integrated education and training programs [10]. To identify key influencing factors and strategies informing future planning and design of the South Australian statewide Foundation Residency program, the study aimed to explore key stakeholders’ expectations and early perceptions of the program structure, role and impact. This study addresses knowledge gaps in evaluating residency program implementation in Australia [10–12] and is framed by an adapted version of Green and Kreuter’s [13] PRECEDE-PROCEED logic model.

## Methods

### Ethics

A written ethics approval for this study was obtained from the Central Adelaide Local Health Network (Quality Assurance study Ref:12429) and the University of South Australia Human Research Ethics Committee (application ID: 202859).

### Design

A qualitative descriptive design was chosen to maintain descriptive and interpretive validity. This was achieved by keeping findings close to the robust data collected, in common language and within an identifiable context [14]. Qualitative descriptive is also an ideal approach underpinning any program evaluation [15]. The COnsolidated criteria for REporting Qualitative research checklist (available as S1 File) will be used to guide study reporting [16].

### Sampling and recruitment

A purposive sampling strategy was adopted to target information-rich participants [15]. Eligible participants were managers of preceptors and residents within the last two years, across various levels of employment within their local health networks. These participants held influence over the program’s implementation, focus and future. To ensure a fair representation, recruitment occurred across all metropolitan and regional pharmacy departments, with each site varying in geography, size, staffing capacity and workflow structures. All participants were recruited directly via email by the lead researcher, that included an information sheet and a consent form. Interested participants voluntarily responded to the invitation by returning a completed consent form and were informed that they can withdraw at any point in time.

### Data collection

Data were collected through individual semi-structured interviews by two independent allied health colleagues external to the program with extensive experience in qualitative research. Interviews were undertaken to capture the distinctive views and perspectives of participants in keeping with the exploratory nature of this research [17]. An interview guide (available as S2 File) was developed by the researchers with the questions informed by previous unpublished preceptor and resident evaluation survey findings. The guide consisted of broad yet comprehensive open-ended questions which explored general perceptions, expectations and experiences of the aims, structures and outcomes of the program. The interview guide was pilot-tested with one of the local health network-based senior pharmacists who was not eligible for the study. This piloting offered insightful feedback. Changes were made to the guide as a result. Each interview was audio-recorded and transcribed verbatim by a professional transcriptionist.

### Data analysis

The transcribed dataset was managed using NVivo software^TM^ (version 10) and was subjected to reflexive thematic analysis (TA) [18]. Thematic analysis enables exploration and description of the dynamic influences and context underpinning phenomenon. Six-phases of reflexive TA were adopted [18]. During data familiarisation, all recording were listened and any biases and assumptions arising from this were documented in a reflective journal to stay true to the data and engage with the data meaningfully. This led to the next phase of systematically going through each transcript to generate codes for each line. Codes were then compared across transcripts to build preliminary themes from the coded data in relation to the research question. The next phase involved maintaining reflexive memos to review thoughts about content and meaning of themes as they continued to develop. Themes were then further revised, defined and renamed to ensure inclusion of all coded data. Data analysis continued throughout the final phase of report writing, which included selected illustrative quotes [18].

### Research rigour

Research rigour was demonstrated through several strategies, addressing four widely adopted criteria; credibility, dependability, confirmability and transferability [15]. The strategies to address credibility included variability across the sample with recruitment from a range of sites and personnel, participant member checking and researcher awareness and documentation of personal biases and assumptions plus maintenance of a reflective journal. The reflexive approach of thematic analysis [18] enabled the first author to reflect on both the strengths of her position as well as the need to actively manage bias. The first author could offer insights and was sensitized to the data through her role in program implementation, program evaluation, leadership roles and senior pharmacist. To engage with the data attentively (dependability) without attaching definitive labels, reflexive memos were used to document thoughts as well as keeping an audit trail to track preliminary thoughts up until final decisions of theme content and meaning. Confirmability was assured using external and professionally trained interviewers who did not know the participants, independent coding of 6 out of the 33 transcripts by other members of the research team and regular research team meetings to identify any discrepancies, discuss findings and their interpretations.

## Findings

A total of 35 participants were invited with 33 consenting to participate. One participant declined citing limited experience with the residency program while the other did not respond within the time frame. Participants consisted of executive/ pharmacy directors, associate pharmacy directors, deputy pharmacy directors (or equivalent), lead pharmacists as well as senior pharmacists overseeing education and training. Participants were randomly assigned a code number with no association to any identifying personal and demographic data. Table 1 outlines an overview of the number of participants at each employment level alongside their concurrent affiliated roles, area of service and location in the state. Other details are not provided to protect participant anonymity. Interviews were conducted in person (n = 29) at a location of participant choosing or via telephone (n = 4) by one of two impartial non-pharmacist allied health colleagues / research assistants across June and July 2020. Each interview lasted approximately 30 to 45 minutes.

**Table 1:** Participant demographics.

Demographic Characteristics		Number (total n = 33)
**Workplace location**	Northern Metropolitan Local Health Network	7
	Southern Metropolitan Local Health Network	7
	Central Metropolitan Local Health Network	10
	Women & Children Metropolitan Local Health Network	2
	Regional Local Health Networks	4
	Statewide South Australian Health Head Office	1
	Statewide Pharmacy Medicines Information Services	2
**Position level**	Executive/ Director of Pharmacy	6
	• makes executive decisions about residency direction at a statewide and local health network level	
	Associate Director of Pharmacy	3
	• weighs in on decisions about residency direction at a statewide and local health network level	
	Deputy Directors (including lead pharmacists)	19
	• represents each local health network as part of the statewide leadership committee, oversees residency implementation operationally at local site; allocates and supports preceptors, some also precept residents	
	Senior Pharmacist	5
	• represents each local health network as part of the statewide leadership committee, implements residency operationally at local site; supports residents and preceptors	
**Area of service**	Clinical	8
	Distributions	5
	Manufacturing	2
	Regional practice	3
	Statewide Medicines Information Service	2
	Executive Management	9
	Education and Training	4

Table 2 outlines the three themes identified that focus on stakeholders’ expectations and early perceptions of the role, structure and vision of the South Australian statewide foundation hospital pharmacist residency program. The PRECEDE-PROCEED model guided the process of highlighting the behavioural, organisational, environmental, and social factors that predispose, enable and reinforce decision-making to progress program implementation [13]. Further data were collected about perceived experiences and visibility of current program implementation, adoption and outcomes and this will be reported elsewhere.

**Table 2:** Overview of themes.

Theme	Overview of Theme Elements
1. Alignment of program goals and visions	Differences and commonality in stakeholder opinion; competing program goals; program recruitment and selection process
2. Culture shift to normalising investing in workforce development	Time, resource and understanding are a barrier; culture shift to residency program as core business; integration of and communication about the program
3. Program structure supports focused workforce development	Bridges training pathway gaps; benefits for whole workforce; implications of rotational structure and statewide model

### Alignment of program goals and visions

This theme describes the misalignment of program goals and visions across stakeholders of the collective statewide public hospital South Australian pharmacy services. This identified need for more alignment of goals and vision signals scope for provision of clear directions from the higher levels of statewide management and residency leadership. The differences in opinion across the participants were a challenge to achieving agreement about future directions and decisions for the program. One participant reflected on this:

*“I think we’ve had difficulties here in this state coming to an agreement between everyone, what that means, and how we translate that into the workplace.” [P26]*

Despite variability in opinions, there was some common ground, with participants acknowledging that the structured developmental pathway streamlined the development of a skilled workforce.

*“for us to have a workforce that further has a set of skills, further educated, further developed, further experiences to be able to operate at a higher level of functioning.” [P12]*

There were three points of commonality amongst the participants. Participants agreed that the rotational structure facilitates transferability of skills through the sharing of knowledge, exposure and experiences across the state. This transferability of skills was perceived to ultimately improve patient care outcomes.

*“you’re trying to develop a better workforce which at the end of the day is for the patients that come into our hospital.” [P1]*

The second point of agreement was the value of some of the residency requirements (such as research), seen to increase interprofessional collaboration. The final point of agreement was participants seeing the program as important quality assurance for staff and services with the potential to *“increase the profile of [statewide organisation] nationally and…internationally*.*” [P18]* The program was also perceived to contribute to workforce retention because *“we bring them into an environment where they’re valued and they’re appreciated…” [P31]*

One of the competing program goals that were debated was whether the vision should be for an all-inclusive program roll-out versus the current exclusive resident recruitment process. Having the program available to all would widen the opportunity for all pharmacists to receive the structured support versus a selective process that limits the intake to only include high performing individuals.

*“I would really like to see that the program was offered to anyone that would like to participate.” [P14]*

The selection process was perceived by participants to be exclusive with concerns raised about creating an elitist workforce. There were references to the initial high performing cohort as well as isolated second-hand perceptions that residents view themselves as superior to non-residents. Exclusivity was also seen to potentially create negativity amongst those that applied to be part of the program but were unsuccessful.

*“Concerns about elitism, … would certain people get opportunities that other people wouldn’t and did I feel comfortable with that? I think because the idea [program] is foundation[al], I found that hard to deal with…” [P17]*

In contrast, there were observations that the limited selection process drove competition and opportunistically recruited motivated candidates. Participants were also concerned that all- inclusiveness would dilute program value and candidate caliber.

“*I don’t think we want to get to a point where everyone goes down the path… there’s nothing special about it*, *if you like*, *everyone might end up with that certification at the end of it*, *that they completed a residency…what’s the deciding factor between people*?*” [P5]*

Another participant was open to the program being used in different ways depending on candidate situation and development needs:

“*you’ve got different benefits…Whether you’re developing someone to really excel and work towards a senior or a specialised position… Or whether you’re allowing staff that … are new to hospital practice to really embed and integrate and develop a knowledge base at a quicker rate” [P30]*

When asked to identify the target cohort for the program, participants saw enrolment as being a personal choice, that acknowledges various contexts and personal circumstances rather than there being a general mandate with no choice.

*“You wanted to make sure that if it’s something we’re rolling out further, that it’s not a forcing function for everybody, because it may not suit everybody to do it.” [P12]*

There were pre-conceptions of significant value for pharmacists who were early career to hospital pharmacy, usually within 1 to 3 years post registration. The benefits were deemed especially profound for pharmacists who did not complete a prior structured hospital internship pathway and for those who had entered hospital practice from a different setting regardless of number of years of practice as a registered pharmacist.

*“It’s a great way for us to help with the training of pharmacists that have come into the hospital system through other avenues. They might come in five, six, seven years down the track, from their registration, but this would still be a great program for them to start in a hospital, which can be quite different from what they’ve already experienced in other areas.” [P14]*

One of the more recent adjustments to program recruitment is that new to hospital pharmacists can only apply upon completion of a certain period of basic orientation training. The decision to make this adjustment has been well-received and aligns with this participant’s view.

*“I think that the residency program has developed …they have to have a bit of hospital knowledge first, which I think is essential because it is a huge thing going into hospital from any other setting and you do need to have your foundation time to do that before you start this sort of intensive assessment thing on top of that…” [P22]*

### Culture shift to normalising investing in workforce development

Some participants identified barriers to their investment in the program and workforce development in general. These included: time and resource commitment for both the residents and preceptors, unclear expectations and the need for further integration and communication about the program to embed a culture of acceptance.

*“I do think it’s some of that cultural stuff … the messaging around the importance and why, because often then people are quick to find reasons not to support things and sometimes if you lose sight of the why, you get lost in the weeds of the logistics … not the bigger picture stuff, which is the end goal.” [P24]*

There also appears to have been a culture shift of awareness towards the importance of prioritising and investing in workforce development as a result of the residency program. Many participants viewed contributing to the professional upskilling of the early career workforce through the residency as core business for the entire statewide workforce.

*“But definitely the intention, the hope is that we actually have a … shift in culture. That providing support to our less experienced pharmacists in our team is part of just everybody’s business … so that people are not left floundering.” [P14]*

*“It’s just the next stage of their development is what I think it should be.” [P21]*

Awareness of the core purpose of the program plays a part towards developing a culture that focuses on workforce development as a priority and as a sustainable investment. Participants shared their perceptions of core values; to improve patient care, to increase workforce quality and capacity, to invest in a workforce that is valued by the organisation thus increasing retention. To achieve these purposes, participants raised the importance of clarity around roles and responsibilities for all involved in the program.

A participant reflected that leadership in decision-making from a management level was pivotal for shifting the gear in culture change toward program expansion: *“I had a discussion with my management team last week and I’m pretty clear with them that we should get to a stage eventually… where everyone does it… it’s more expected they participate*.*” [P24]* Similarly, another participant suggested the possibility of expanding the workforce development program beyond the early career pharmacists to include pharmacy assistants and senior pharmacists: “*But can the program—is there plans to roll it out*? *Not just for the junior pharmacist*, *but is it going to be a similar program for all of the workforce*?*” [P10]*

Several participants raised observations of comparison between the local statewide program with other national and international pharmacy residency models as well as the medical model of career progression. They saw a need to adopt a similar ‘business as usual’ culture in South Australian hospital pharmacy that promotes an adaptable and contemporary workforce.

*“from an academic point of view or professional development point of view it’s perfectly aligned with what other countries offer, so I think it’s very important to have this program … in our workforce, to align with contemporary offers.” [P8]*

Participants clearly reiterated that program uptake is dependent on all parties engaging and investing in the program, across all levels of involvement. This applies to residents embracing a culture of being informed, *“…I would sincerely hope that they would read through the job pack properly and understand what it entails*, *rather than entering the program and then dropping afterwards for whatever reason*.*” [P4]* For preceptors, this is dependent on having realistic expectations around their respective roles and responsibilities: *“Some preceptors probably going in think[ing]*, *it’s too much hard work*, *because I have to catch up with them all the time*. *But it’s actually not any different to teaching on the run [embedding teaching moments in practice]*.*” [P6]* For the overall workforce, participants felt a need for understanding of organisational expectations for addressing their core development needs.

Acknowledging that culture change is a gradual process, further integration of the program into current workflows is required. Better communication of program structure and outcomes to the pharmacy managers would support overall awareness and program advocacy.

*“…the message is clearly not getting through [to] me in a way that means something to me.” [P33]*

### Program structure supports focused workforce development

The program structure is key to success in focused development for residents and potentially for the rest of the workforce. The residency is viewed as a bridge over a gap in training pathways for the early career and early to hospital pharmacists. Beyond the already established pharmacy internship program, there is a perceived lack of structured and consistent workplace developmental pathways prior to the residency program construct.

*“There’s always been a very structured intern program for our new pharmacists…and there was a gap afterwards… It was almost like a year where people would be really supportive, and then it was let loose in the wild worlds of pharmacy… Similarly, for people who worked in community, when they came to hospital, they initially had periods of support, it’s development but then there was no real support and oversight. So, for me that’s really where the residency fits in…” [P26]*

The structure effectively enforces learning opportunities through intentional self-reflection, regular mentor feedback and focused development through the practice-integrated structured assessments, research and post-graduate components. Some of the residency assessment tools have also been used to support non-residents, further extending the influence of the program structure.

*“I think in having those structures, everybody sort of comes along with that journey, even if you’re not a resident… the advantage is it pushes the individual to seek those opportunities out…” [P17]*

As a result, the residency structure appears to promote a culture of shared teaching and learning through *“…osmosis…” [P6]* whereby preceptor and resident mentoring occurs through *“…learning to give feedback…” [P26]*. Visibility of this teaching and learning environment also motivates non-residents to be involved and in turn *“…all the team is lifted up somehow…” [P8]*

Participants highlighted that the credibility of the program is strengthened by adopting the SHPA-accredited framework model. Unique to Australia, the South Australian program is the only statewide-run and governed residency program. The program offers strategic exposure and upskilling through the opportunity to rotate to various practice areas across metropolitan and regional settings and draws its strengths from being supported through a statewide service framework and training resource.

*“[South Australian statewide public hospital pharmacy service] has been incredibly supportive with offering various types of workshops…offering all that sort of opportunities for the resident…I think certainly would steer the outcome…in the positive direction…… Remembering, as well, that the residency program is also adapted from SHPA, so all of the tools that we have all came from SHPA…And we’ve set up the state-wide support network… has been very beneficial for all of us. There is also a residency leadership group.” [P4]*

Interestingly, the residency has broken down barriers to staff movement across sites which was perceived as uncommon across Australia, and thus builds the ability to flex and cover where needed due to working knowledge of different parts of the service.

*“We’re siloed at sites sometimes, we’re also siloed still within the regions, but the residency program has really broken that open so that people go to any region.” [P5]*

*“…pass on some of our learnings that they can go out and take to their own sites as well… if we have gaps in our staffing, we can now draw on some other people that have the skills to come in to help us out as well… more consistency across the board…” [P1]*

The fixed rotations were frequently mentioned, appraised as an enabler to providing the residents with an essential and well-balanced exposure to four core foundational areas i.e. medical, surgical, operational and a suitable strategic area. P4 and P6 affirmed that the program and rotation duration, 2 years and 6 months respectively, enable adequate exposure.

“*And so I think in terms of the pillars or the structure of the program*, *the fixed rotations*, *which gives the pharmacist a core understanding in those really core clinical pharmacy areas*, *so the surgical and medical rotations for instance*, *coupled with that constant feedback loop is what makes the program successful” [P15]*

Conversely, the rotations were seen as a barrier to expansion of the program due to the *“practicalities of the rotations” [P34]* and *“…they’re quite rigid…potentially limiting growth” [P14]* Barriers to the cross-site rotations focused on additional efforts to streamline daily operational and logistical challenges. These challenges were particularly observed to arise from staffing gaps due to a premature exit of a resident from the program, time spent to provide orientation, differences in staffing resource allocation across the statewide service as well as concerns around a correct balance of skill mix to flexibly integrate a resident rotating from a different local health network in an area of need.

***“**The other cons are how we …incorporate a resident who may be rotated to different hospitals? So, if the resident rotates to one hospital, and then we get someone else here, do they have the right skill mix for where we need[cover]?” [P10]*

Regular movements across the rotation sites were also seen to hamper continuity and timely completion of the research project, creating challenges for residents and supervising preceptors.

*“…the rotational nature of the residency…doesn’t particularly lend itself very well to a research project… so it’s difficult then to coordinate the continuation of that project…with the research project running over full two years, whilst the rotations are only every six months.” [P15]*

In contrast, a participant reframed this perceived problem in terms of considering the service as a collective *“…we all need to think of us as SA [South Australian] Pharmacy*, *not as individual sites*. *And that if we’re committing to this that there needs to be an understanding that all the residents will rotate…*.*” [P6]* A recent adjustment to the number of cross-site rotations to address the above challenges was favourably noted *“But from this year we’ve reduced the [cross-site] rotation to one instead of two…Two was too much*.*” [P3]*

Rural and regional rotations have also been raised as a point of contention. There are two different perspectives; a regional rotation is integral as it provides a unique generalist experience *“…for a residency program which is promoting general experience*, *I think the country sites have a lot to offer*.*” [P29]* versus views about the organisational logistical and training resource limitations in regional areas “*… we don’t run like a metropolitan hospital*…*So*. *the intention of having a resident is that I don’t have to train them*, *they already have the basic knowledge*, *I just have to teach them the local differences of what happens in country*.*” [P13]* as well as an understanding that residents may have competing life circumstances which discourages this as a forcing function *“… it’s a bit more challenging when you’ve got to change your life for six months of the year and go elsewhere…” [P2]*

## Discussion

There is limited research evaluating the implementation of practice-integrated education and professional development programs across Australia. This gap in knowledge is likely due to the historical context of there being no structured practice-integrated pathways in pharmacy post-registration prior to the nationwide rollout in 2016. In a historical sense, the SHPA residency program is still in its early days, with only 63 out of the total 300 resident pharmacists having completed the 2-year program [12]. As one of the first SHPA-accredited sites, Lui, Toner [11] published findings from an evaluation of their foundational pharmacist residency program implemented at a single-center metropolitan tertiary hospital in Victoria, Australia. In 2019, SHPA as Australia’s sole residency program accreditation provider, surveyed the graduated resident cohort nationwide and their program managers. Out of the survey, 64% of residents perceived that the program had improved career prospects and 79% of program staff perceived implementation to be difficult [12]. Our research has explored stakeholder expectations and early perceptions of the role, structure and vision of a novel statewide early career hospital pharmacist foundation residency program in South Australia for the purposes of informing future focus and refinement. A unique feature of the program lies in its multicentered model which also incorporates an integrated university-affiliated post-graduate course. The program in its current form has workforce resource implications and is often under scrutiny to evaluate if its clinical and workforce outcomes justify the investment input [19]. Using an adapted version of Green and Kreuter [13] PRECEDE-PROCEED framework, this study found three themes which included the need to align goals and vision, facilitate a culture shift toward investing in workforce professional development and implementation of a program structure that supports and directs workforce development.

There were varying perspectives about program goals and visions amongst the stakeholders. Commonly agreed upon aims included to strategically create an upskilled workforce with transferable skills across the statewide service, to develop well-rounded and motivated health professionals, to improve patient care outcomes, to increase pharmacy presence through interprofessional engagements and to increase staff retention. Common timelines for program entry are consistent with 1 to 3 years of hospital practice as defined by SHPA although some acknowledged that number of years may not be a definitive factor if individuals seek the development opportunity. Collectively, participants advocated for an optional and voluntary program entry with some intensive orientation and basic training prior to residency entry. Wang, Clavarino [10] affirms that to meet contemporary workforce needs, the residency program needs to integrate its objectives parallel to existing aims in hospital pharmacy education and training. Health workforce investment as well as, foundation training and early career development, is a clear collective objective on a global scale [1,20].

On competing program goals, views differed from being a ‘business as usual’ foundational skill-development program to support all early career pharmacists to being a special elite program that selectively supports higher performing pharmacists towards senior role reclassifications. Similar concerns of inequity between residents and non-residents were also reported across other Australian residency sites [10]. However, some participants are in favour of the current number-limited merit-based recruitment to uphold program credibility and resident calibre. SHPA, the accrediting body, deliberately provides a generic vision, framework and overarching program structure to be tailored at the implementing sites. Hence, the broad directions may result in differences in expectations, experiences and implementation across the sites statewide [10]. The differences in understanding of program purpose and scope were a perceived challenge to achieving consensus for future directions. More streamlined direction and clearer expectations at a statewide senior level [10,21,25], was viewed as an enabler to guide future program goals [26]. Similar dilemmas are seen globally where the ideal vision for all-inclusiveness [2,21,22] does not match some of the existing selective recruitment residency models. This barrier stems largely from resource and vacancy limitations [21–24] with a need for an expansion of site capacity to occur [19].

Interestingly, the statewide residency program appears to have already initiated a culture shift of awareness towards prioritising and investing in workforce development. Many participants viewed contributing to the professional upskilling of the early career workforce as an expected core business and a natural career progression for the entire workforce [10]. Further integration of the program into current workflows was seen as necessary to normalise and maintain this new culture. Wang, Clavarino [10] view that residency is yet to be fully embraced as a practice norm across Australia, jeopardising sustenance and further program establishment. This is consistent with Green and Kreuter [13]’s assessment of behavioral patterns that either protect or risk planning success. Implementation of the program has prompted a shift away from junior staff being under equipped due to a lack of structure and consistency for support across the statewide hospital pharmacy service. This shift aligns with several aspects; expectation of a structured professional development continuum within a safe and supported environment on par with contemporary intra and interprofessional models [26], global health workforce scaling up priorities and evolving global capacity challenges [1,4].

As part of an educational and ecological assessment [13], predisposing factors that play a part in shifting the workforce towards a culture of prioritising workforce development include an awareness of potential program core benefits as well as clarity around roles and responsibilities for all involved. The perceived key core benefits justifying investment include; improved patient care as the ultimate goal [4], increased workforce quality and capacity [4,25], a shared teaching and learning environment [25], reflects a workforce valued by the organization with increased workforce retention. The program speaks for itself through enabling [13] transferability of experiences and skills. Observability of resident involvement and associated advantages also reinforces and motivates participation from the entire workforce [10,13]. As for roles and responsibilities, participants unanimously agreed that the residents need to be fully aware and informed of the program expectations and requirements in order to truly commit [27]. Resident motivation is also perceived to influence preceptor engagement, while acknowledging that the preceptor’s role is predominantly supportive and should fit into a sustainable education model [25]. Support is needed at all levels of the organisational structure [19] including at a pharmacy management and program director level in order to spearhead a culture change that advocate for time dedicated to workforce development [25]. One of the gaps raised was around communication of program value from the program coordinators to the managers. Zellmer [26] encourages system executives to be abreast of program value as a key to program growth. Change management is acknowledged to be a gradual process and involves a concerted effort [27].

The findings from this research also clearly identified the residency program structure and core elements as vital and modifiable environmental factors influencing program planning [13]. The developmental structure afforded to the residency is perceived to bridge a historical gap in the pre-existing training pathways for the early to hospital career pharmacists, reflecting similar perceived program roles across other countries [22–24]. The consistent set of core rotations was frequently raised as an enabling factor [13], seen to effectively consolidate and promote workforce skillset transferability through focused exposures across different practice areas, settings and health networks. The other program structure elements feature the practice-integrated assessment tools, research [11] and the postgraduate course. These elements were perceived to facilitate and enforce intentional self-reflection and mentoring. Many viewed clarity of structure as the key supporting pillar of program implementation; a view which draws its credibility and consistency from adopting a national accredited model [5,25] mapped against the national pharmacy competency standards [11] and from being coordinated as a statewide program entity.

The streamlined and targeted development pathway upskills and supports the early to hospital career cohort [5,10,25] as well as the rest of the workforce. The subsequent increase in workforce capacity lends itself as an enabling factor towards program expansion. Conversely, the rotational structure was also viewed as a barrier to program expansion, thus creating a point of contention amongst the stakeholders. A key barrier presents itself in the form of operational and logistical challenges centred around the coordination of the rotation schedule across the statewide program [5,25], including comments that highlighted the benefits versus limitations of a rural and regional rotation. Winegardner, Davis [5] and Vong, Koons [25] reiterate that effective communication and coordination between the residency program leads and all participants are imperative to minimise disruptions and duplication of efforts, and to keep priorities focused on the residents’ education and training.

### Strengths and limitations

The strength of this study lies in the large sample size, use of independent external interviewers, as well as a structured interview guide that was piloted and refined consistent with best practice standards [16]. However, limitations of this study must also be acknowledged. Residents and preceptors were not included in the study because their views are captured through regular prospective longitudinal online surveys and the aim of the research was to explore perspectives of the leadership team. The findings of the surveys informed development of the interview questions. A small percentage (12%) of participants were interviewed via telephone due to physical locations and convenience, hence creating a risk of communication gaps from the absence of non-verbal cues. As it operates statewide, the foundational residency programs differ in structure and process across different sites and hence this program may not be directly transferrable to other contexts.

### Implications to practice and research

This research, has shed light on perspectives about common and competing views of key stakeholders about the residency program role, its structure and vision. Overall, these findings suggest that incremental increases in program capacity will occur over time, as culture changes. It is recommended that focus be placed on supporting and sustaining the change in culture that has begun and that there be continued investment in workforce development. Furthermore, it advisable that the core program purpose and workforce expectations be established with the view to the hospital pharmacy foundation residency program becoming core business. Doing so, will increase resource capacity and enable structural and recruitment flexibilities. While this study has addressed an important knowledge gap, ongoing research is required to strengthen the evidence base for residency programs across disciplines [28]. For example, a systematic review could map and summarise global literature reporting on similar practice-integrated professional development programs or frameworks for early career hospital pharmacists, their evaluations, and findings. Similarly, longitudinal research could be undertaken to investigate the impact of pharmacy foundation residency programs on the pharmacists, organisation and the health system more broadly.

## Supporting information

S1 File COREQ (COnsolidated criteria for REporting Qualitative research) checklist. (PDF)

S2 File SA Pharmacy foundational residency program formal evaluation. Interview guide and data collection form.
(PDF)
