# Supplementary material for: “Trying to develop a better workforce”: Stakeholders’ perspectives of a practice-integrated Australian hospital pharmacist foundation residency program
Source: PLoS One. 2022 Jun 21;17(6):e0270051. doi: 10.1371/journal.pone.0270051 (PMC9212144; doi:10.1371/journal.pone.0270051)
Supplement: S2 File — Interview guide and data collection form. (PDF) [file pone.0270051.s002.pdf]

Appendix

Interview guide and data collection form

SA Pharmacy Foundational Residency Program: Formal Evaluation

INTERVIEW GUIDE AND DATA COLLECTION FORM

**Name of Participant**	
**Position Title**	
**Local Health Network**	

**Date of Interview**	
**Time of Interview**	
**Venue of Interview**	

Part 1 – Establish Participant Understanding and Confirmation of Consent

This interview will take place with the above-named participant, currently one of the SA Pharmacy key stakeholders directly or indirectly managing preceptors and/ or residents involved in the SA Pharmacy Foundational Residency Program.

Explain the aim of the study; Primary aim is to identify the barriers and enablers to allow for further expansion of the state-wide Foundational Residency Program. Secondary aim is to explore the expectations, experiences and perceptions of the role and structure of the state-wide program.

Provide the 'participant information sheet and consent form'.

Enrolment checklist:

- Ensure that the participant is given the opportunity to read and understand the information sheet, as well as ask any questions to clarify
- Ensure the consent form is signed by the participant to be interviewed and audio recorded
- Ensure the original copy of the signed final 'participant information sheet and consent form' is given to the participant, a copy of the signed consent form is retained and provided to one of the study investigators.

Part 2 - Interview

Good morning/afternoon Mr/Mrs/Ms ______________________________

Thank you for agreeing to participate in the SA Pharmacy Foundational Residency Program: Formal Evaluation and for agreeing to answer a few questions.

May I please confirm whether you are still willing to participate in this study?

These questions should take no more than thirty minutes. You can make extra comments about any of these questions if you want, and I'll write those on the form next to your answers. We will be recording this interview, so that I can record all of your answers accurately.

Are you ready to begin?

1. What is your understanding of the SA Pharmacy Foundational Residency Program?
2. What are your general perceptions about this program? What do you think about it?
3. How do you feel this Program is influencing your workforce? Pharmacists development of competence (both residents and preceptors), employment prospects, career trajectories

4. How do you feel the structures making up the program are influencing the outcomes? (i.e. rotations across LHNs, learning tools available, research project, having designated preceptors, workshops and extra learning opportunities)
5. In an ideal world, how would you like this foundational residency program to work?
6. What outcomes do you notice that can be attributed to this Program? (positive or negative) – not just for the residents but also for the preceptors, the departments, the LHNs …?
7. Do you have early career hospital pharmacists (first two years) that are not part of the residency program and how does their performance differ from the resident early career pharmacists? Who do you believe this program should be offered to?
8. In 2019, the program was offered to and taken up by 14/45 early career hospital pharmacists. What are your thoughts around rolling this Program to include all early career hospital pharmacists? Potential barriers? Potential enablers?
9. Do you have any other thoughts about this foundational residency pharmacy program that you have not had opportunity to share so far?

Probing questions that may be needed to explore points in more detail: Can you tell me more about that? What do you mean by …? How does that make you feel?

This concludes the interview. Thank you for your time.

Interview conducted by: Name ____________________________

Signature_____________________________

Interviewer comments (if any)

………

………

………

………

………

………

………

………

………

………

…………………………………………
